# PDLIM5 Affects Chicken Skeletal Muscle Satellite Cell Proliferation and Differentiation via the p38-MAPK Pathway

**DOI:** 10.3390/ani11041016

**Published:** 2021-04-04

**Authors:** Haorong He, Huadong Yin, Xueke Yu, Yao Zhang, Menggen Ma, Diyan Li, Qing Zhu

**Affiliations:** 1 Farm Animal Genetic Resources Exploration and Innovation Key Laboratory of Sichuan Province, Sichuan Agricultural University, Chengdu 611130, China; hehaorong@stu.sicau.edu.cn (H.H.); yinhuadong@sicau.edu.cn (H.Y.); yuxueke@sicau.edu.cn (X.Y.); zhangyao@sicau.edu.cn (Y.Z.); diyanli@sicau.edu.cn (D.L.); 2 College of Resources, Sichuan Agricultural University, Chengdu 611130, China; mgen@sicau.edu.cn

**Keywords:** skeletal muscle satellite cells, PDLIM5, p38-MAPK pathway, proliferation, differentiation, chicken

## Abstract

**Simple Summary:**

PDZ and LIM domain 5 (PDLIM5) can increase C2C12 cell differentiation; however, the role of PDLIM5 in chicken skeletal muscle satellite cells (SMSCs) is unclear. In this study, the effect of PDLIM5 was verified on SMSCs in vitro, and then the molecular mechanism was determined by transcriptome sequencing. We demonstrated that PDLIM5 can positively affect chicken SMSC proliferation and differentiation via the p38-MAPK (mitogen activated kinase-like protein) pathway. These results indicate that PDLIM5 may be involved in chicken skeletal muscle growth and development.

**Abstract:**

Skeletal muscle satellite cell growth and development is a complicated process driven by multiple genes. The PDZ and LIM domain 5 (PDLIM5) gene has been proven to function in C2C12 myoblast differentiation and is involved in the regulation of skeletal muscle development. The role of PDLIM5 in chicken skeletal muscle satellite cells, however, is unclear. In this study, in order to determine whether the PDLIM5 gene has a function in chicken skeletal muscle satellite cells, we examined the changes in proliferation and differentiation of chicken skeletal muscle satellite cells (SMSCs) after interfering and overexpressing PDLIM5 in cells. In addition, the molecular pathways of the PDLIM5 gene regulating SMSC proliferation and differentiation were analyzed by transcriptome sequencing. Our results show that PDLIM5 can promote the proliferation and differentiation of SMSCs; furthermore, through transcriptome sequencing, it can be found that the differential genes are enriched in the MAPK signaling pathway after knocking down PDLIM5. Finally, it was verified that PDLIM5 played an active role in the proliferation and differentiation of chicken SMSCs by activating the p38-MAPK signaling pathway. These results indicate that PDLIM5 may be involved in the growth and development of chicken skeletal muscle.

## 1. Introduction

The largest edible part of meat animals is muscle tissue. Muscle is composed of muscle cells (also known as muscle fibers) that can be segmented into skeletal muscle, myocardium, and smooth muscle. Among them, skeletal muscle is regarded as the most abundant, accounting for 45–60% of an adult animal’s body weight [1,2]. In livestock and poultry, the quantity and quality of skeletal muscle directly influence meat production and determine the economic value of animals. After the postnatal period, animal skeletal muscle development primarily relies on the activation of skeletal muscle satellite cells (SMSCs) to form new muscle fibers [3,4] while the number of fibers are basically consistent. These complex processes are controlled by regulatory factors, including a set of genes and signaling pathways [5].

PDZ and LIM domain 5 (PDLIM5) is a member of the PDZ and LIM domain family, and is also known as Enigma homologous protein (ENH). The PDZ and LIM family is characterized by containing an N-terminal PDZ domain and one or more C-terminal LIM domains [6]. The PDZ and LIM domain plays a non-negligible role in various biological processes by encoding a number of proteins. As one among them, PDLIM5 is linked to a number of medical problems, including mental illnesses [7] and heart diseases [8], and its expression has caused concern with the progression of gastric cancer [9], lung cancer [10], and breast cancer [11]. The PDZ domain consists of 80–90 amino acids and is one of the most common binding domains. The mode of action of the PDZ domain is to interact with the C-terminal or internal binding site on the target protein. It can be combined with actin-binding proteins α-actin, palladin, or β-tropomyosin to participate in the process of myogenesis. The LIM domain generally consists of 50–60 amino acids and is characterized by the inclusion of two zinc finger structures. Similar to the PDZ domain, it is highly conserved. The LIM domain can bind to a diverse range of target proteins, from signal molecules to some transcription factors, including actin and cytoskeleton components. Therefore, in addition to participating in muscle development, its biological functions are more complicated, and it is also expressed in the brain, liver, and bones [12,13]. Currently, there are 10 genes identified in the PDZ and LIM domain family. Studies have shown that all PDZ and LIM domain-encoded proteins can bind to the actin cytoskeleton or affect the cytoskeleton, and several proteins are mainly expressed in skeletal muscle [14]. Previous research has reported that PDLIM5 is highly expressed in mice skeletal and heart muscle, and that it functions as a signal modulator to influence muscle tissue development [15]. However, the underlying mechanism of PDLIM5 on chicken skeletal muscles remains unknown. Mice are a model animal for mammals, and the study of PDLIM5 in mouse skeletal muscle is of little relevance to the study of chicken myogenesis. What effect PDLIM5 has on chicken muscle development is worthy of further research. This can also provide a more valuable reference for the study of PDLIM5 in avian myogenesis, even including the function of PDZ and LIM domain family genes.

SMSCs can also be called adult myoblasts, which are a special type of myoblasts. Skeletal muscle satellite cells play a key role in maintaining skeletal muscle growth and muscle fiber regeneration after injury. Compared with other muscle-derived cells, a remarkable feature of SMSCs is that they can undergo self-renewal through symmetrical and asymmetrical division [16,17].

In this study, we verified the effects of PDLIM5 in chicken SMSCs and found that PDLIM5 plays a positive role in the proliferation and differentiation of SMSCs. Additionally, through transcriptome sequencing, the results show that KEGG enriched the p38-MAPK signaling pathway after interfering with PDLIM5 and further confirm the molecular mechanism of PDLIM5 regulating chicken myogenesis through the p38-MAPK signaling pathway.

## 2. Materials and Methods

### 2.1. Ethics Approval

Chicks were given enough feed and water (twice a day) and cages were regularly cleaned. Temperature was kept at around 34 °C, and it was adjusted at any time according to the chicken’s condition and the outside temperature to ensure its normal development. In brief, all content complied with the approval of the Animal Ethics Committee in Sichuan Agricultural University (2019–0717).

### 2.2. Animals and Cell Culture

In this study, the breast muscles of four-day-old ROSS 308 broilers were collected to extract SMSCs. The specific steps of SMSC collection were detailed in a previous study [18]. The cell cultures in the experiment required two media. A growth medium (GM) used to promote cell growth and proliferation was composed of 89% Dulbecco’s modified Eagle’s medium (DMEM; Gibco, Grand Island, NY, USA), 1% penicillin/streptomycin (100×) (Invitrogen, Carlsbad, CA, USA) and 10% fetal bovine serum (Gibco, New York, USA). A differentiation medium (DM) used to induce cell differentiation was obtained by mixing 98% DMEM and 2% horse serum. We first use the GM to culture the SMSCs to a cell confluency of 80–90% and then changed to the DM to induce the differentiation of SMSCs. The cell incubator provided a constant temperature (37 °C) and constant carbon dioxide (5% CO_2_) level for cell growth. Eleven different types of tissue were collected from four-day-old ROSS 308 broilers, namely, heart, liver, spleen, lung, kidney, pectoral muscle, leg muscle, brain, fat, intestine, and gizzard tissue. After grinding in liquid nitrogen, RNA was extracted from the 11 tissues.

### 2.3. Vector Construction, RNA Oligonucleotides, and Cell Transfection

Lipofectamine 3000 (Invitrogen, California, USA) is a liposome that helps siRNA (Small interfering RNA) or plasmid pass through the cell membrane and enter the cell. The Opti-MEM (Gibco, New York, NY, USA) and Lipofectamine 3000 were used to transfect cells. We conducted transfection when the cell confluency of SMSCs reached about half, and we explored the effect of siRNA or plasmid on cell proliferation. When the cell confluency reached 80–90%, transfection was performed to study the effect of siRNA or plasmid on cell differentiation.

The PDLIM5 siRNA and overexpression plasmid were designed according to the PDLIM5 sequence on NCBI (accession number: NM_001031149.2), as shown in Table 1. The siRNA of PDLIM5 was purchased from GenePharma (GenePharma, Shanghai, China). The coding sequence (CDS) of PDLIM5 was amplified from chicken genomic DNA by the polymerase chain reaction (PCR) method. Using T4 DNA ligase, the PDLIM5 coding sequence fragment was digested with restriction enzymes and ligated to the pcDNA3.1(+) vector. After verification, the successfully constructed vector was named pcDNA3.1-PDLIM5, and the overexpression plasmid was constructed by Sangan Biotech (Shanghai, China).

### 2.4. RNA Extraction and Expression Detection

According to the instructions of the reagent, TRIzol reagent (Invitrogen, California, CA, USA) was used to extract total RNA. After extracting, integrity and concentration of RNA in samples were measured using a Thermo Scientific™ NanoDrop Lite (Thermo, Waltham, MA, USA). After determining the integrity and concentration of RNA to meet the conditions (2.0 > OD_260/280_ > 1.8, OD_260/230_ > 2.0), a PrimeScript RT Master Mix Perfect Real Time (Takara, Dalian, China) was used for reverse transcription. Real-time PCR primers were designed using Primer Premier 6 and are listed in Table S1. The reaction volume for real-time PCR was 10 μL and consisted of 1 μL cDNA, 0.5 μL reverse and forward primers (per gene), 3 μL double-distilled water, and 5 μL TB Green™ Premix Ex Taq™ II (Takara, Dalian, China). Finally, a real-time PCR detection system (CFX96-Touch; Bio-Rad, Hercules, CA, USA) was used to determine the gene expression. In this study, β-actin was used as an internal reference gene. The 2^−ΔΔCt^ method was used for data analysis.

### 2.5. CCK-8 and EdU (5-ethynyl-2′-deoxyuridine) Assay

Cell Counting Kit 8 (CCK-8; Meilunbio, Shanghai, China) was used to detect cell proliferation. After 12, 24, 48, and 72 h of transfection, 10μL CCK-8 reagent was added to each well of a 96-well plate, and the absorbance was measured after incubating for 1 h at 37 °C. The C10310 EdU Apollo in vitro imaging kit (RiboBio, Guangzhou, China) was used for the EdU experiment. The specific reaction of EdU and Apollo fluorescent dyes was used to quickly and accurately detect the proliferation ability of cells. Three areas were selected randomly to evaluate the mount of stained cells. The detailed operation steps for EdU and CCK-8 tests were outlined in our previous research using the EdU test and CCK-8 test kits [18].

A BD AccuriC6 flow cytometer (BD, Franklin Lakes, NJ, USA) was used to measure the cell cycle of SMSCs, and the measured cells were suspended overnight in 75% ethanol the day before. Before the determination, a propidium iodide/RNase staining buffer (BD, Franklin Lakes, NJ, USA) was added and incubated at 37 °C for 15 min. Finally, the software of Modfit LT 3.2 (Verity Software, Topsham, ME, USA) was used for subsequent analysis.

### 2.6. Immunofluorescence and Western Blot

The immunofluorescence and Western blot were detailed in our previous study [19]. Following the instructions, the cells were collected when they differentiated into obvious myotubes, and 4% paraformaldehyde (Solarbio, Beijing, China) was used to fix the cells. Subsequently, an immunofluorescence test was performed, and three pictures were taken randomly each time. A fluorescence microscope (Olympus, Tokyo, Japan) was used to take pictures, and the professional software of Image-Pro Plus 6.0 (Media Cybernetics, Bethesda, MD, USA) was used to measure the myotube area.

The cells were collected 72 h after transfection. The protein was extracted with a lysis buffer, and the protein concentration was determined using a bicinchoninic acid (BCA) protein detection kit (Beyotime, Shanghai, China). The total protein was separated on SDS-PAGE, and then the PVDF (polyvinylidene fluoride) membrane was incubated with the primary antibody overnight. Incubation with the secondary antibody was performed for 1 h at room temperature the next day. Finally, an enhanced chemiluminescence (ECL) luminescent fluid (Solarbio, Beijing, China) was used to detect the antibody response band. β-Tubulin was used as an internal control, and the secondary antibody was horseradish peroxidase (HRP) labeled anti-mouse immunoglobulin G (IgG) (ZenBio, Beijing, China; 1:2000). Table S2 shows the antibody information used in the Western blot experiment.

### 2.7. Transcriptome Analysis

After the cells grew to a density of 60–70%, SMSCs were transfected with si-PDLIM5. After 48 h, total RNA was isolated from si-PDLIM5 and normal cells. Only high-quality RNA samples (2.0 > OD_260/280_ > 1.8, OD_260/230_ > 2.0) were sequenced through Beijing Novogene Technology Co., Ltd. (Beijing, China) for transcriptome sequencing. The original data were uploaded to the GEO (Gene Expression Omnibus) database (NCBI accession number: GSE151450). DESeq was used to analyze the difference in gene expression and screen the differentially expressed genes as expression difference times| log2 times change|> 2, significant *p* value < 0.05.

### 2.8. Statistical Analysis

The statistics software of SPSS 17.0 (SPSS, Inc., Chicago, IL, USA) was used in this study. All data are shown as the least squares means ± standard error of the mean (SEM). The unpaired Student’s *t* test and one-way ANOVA were used to compare two or more groups, respectively. Before conducting multi-group difference analysis, a normality test should be carried out first and it can be carried out only after meeting the requirements. Differences were considered significant for all statistical tests at *p* <0.05.

## 3. Results

### 3.1. PDLIM5 Tissue Distribution in Vhicken

**Figure 1:**
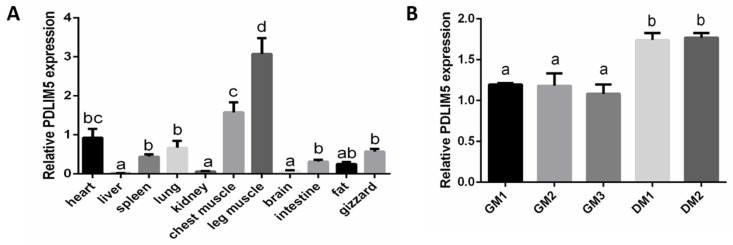
The expression of PDZ and LIM domain 5 (PDLIM5) in different chicken tissues and chicken skeletal muscle satellite cells (SMSCs). (**A**) The expression of PDLIM5 in 11 different chicken tissues determined by qPCR (heart as the control group, *n* = 9). (**B**) The expression of PDLIM5 in chicken SMSCs at different time points determined by qPCR (*n* = 3). ^a,b,c,d^ *p* < 0.05.

First, we detected the PDLIM5 expression in 11 different chicken tissues. Based on the result of real-time PCR, we found that the PDLIM5 gene was more highly expressed in chest and leg muscle and the heart among the selected 11 different tissues used (leg muscle, chest muscle, heart, spleen, gizzard, liver, lung, kidney, intestine, abdominal fat, and brain) (Figure 1A). This result indicates that PDLIM5 is more expressed in the development of chicken heart and skeletal muscle. We then used chicken SMSCs as a model to identify the functional characteristics of PDLIM5 in skeletal muscle. The results show that the differentiation of SMSCs began on the fourth day, and the expression of PDLIM5 was significantly up-regulated during cell differentiation (Figure 1B).

### 3.2. PDLIM5 Increases Proliferation of Chicken SMSCs

**Figure 2:**
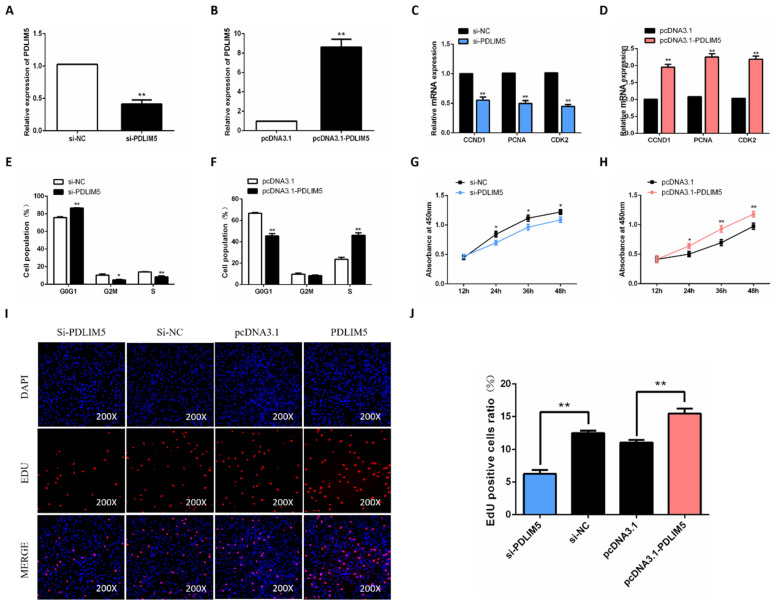
PDLIM5 increased proliferation of chicken SMSCs. The mRNA expression of PDLIM5 in cells that transfected with si-RNA (**A**) or plasmid (**B**). After 48 h of PDLIM5 inhibition (**C**) or overexpression (**D**), qPCR was used to detect SMSC proliferation-related genes. Cell cycle analysis of SMSCs after inhibition (**E**) or overexpression (**F**) of PDLIM5 for 48 h. (**G**,**H**) After overexpressing PDLIM5 or inhibiting PDLIM5 in SMSCs, the cell proliferation activity was measured using CCK-8 kit. (**I**,**J**) The EdU fluorescence (red) was considered the proliferation indicator, and the cell nuclei were represented by DAPI (4’,6-diamidino-2-phenylindole, blue) fluorescence. The ratio of EdU-positive cells was determined when PDLIM5 was overexpressed or inhibited. * *p* < 0.05; ** *p* < 0.01 vs. NC and pcDNA3.1 (negative control). (*n* = 9).

To evaluate the function of PDLIM5 in chicken SMSC proliferation, the effective plasmids and PDLIM5 siRNA were constructed to overexpress and knock down PDLIM5 in cells. As a result, after adding the PDLIM5 overexpression plasmid to SMSCs, the expression of PDLIM5 increased by 8.6 times. After knocking down, the expression of PDLIM5 decreased by 0.41 times. The results show that the interference and overexpression efficiency is significant, which meets the subsequent experimental requirements (Figure 2A,B). Subsequently, we tested the mRNA expression of cyclin D1 (CCND1), cyclin dependent kinase 2 (CDK2), and proliferating cell nuclear antigen (PCNA), and found that overexpression of PDLIM5 effectively increased the abundance of these genes (Figure 2D). At the same time, cell cycle analysis showed that in PDLIM5 overexpressed cells, the cell population increased in S and G2/M phases but decreased in G1/0 phase (Figure 2F), and the cell activity also increased (Figure 2H). Meanwhile, knocking down the expression of PDLIM5 in SMSCs had the opposite effect. (Figure 2C,E,G). After adding si-PDLIM5, the number of EdU staining positive cells and the cell survival rate were lower than those in the control group, while adding pcDNA3.1-PDLIM5 to SMSCs showed the opposite (Figure 2I,J). These results indicate that PDLIM5 promotes SMSC proliferation.

### 3.3. PDLIM5 Increased Differentiation of Chicken SMSCs

**Figure 3:**
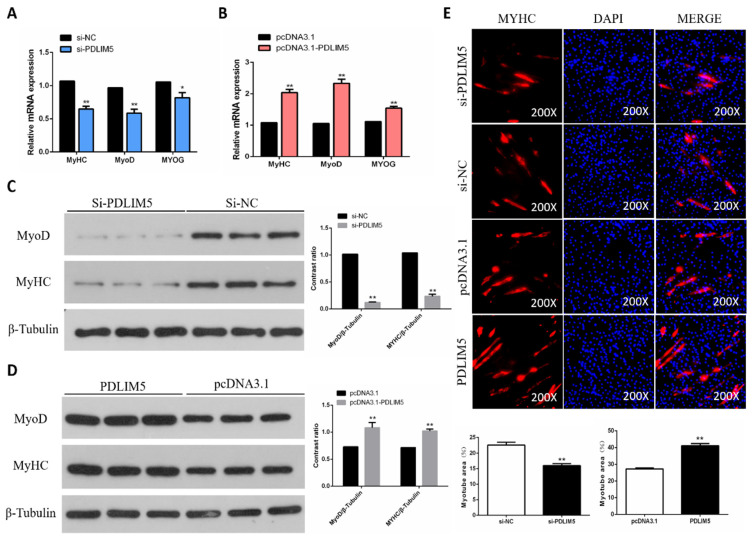
PDLIM5 increased chicken SMSCs differentiation. (**A**,**B**) Gene expression of myosin heavy chain (MyHC), myogenic differentiation 1 (MyoD), and myogenin (MyoG) in SMSCs after overexpression or knockdown of PDLIM5. (**C***,***D**) Protein abundance of MyHC and MyoD in SMSCs after overexpression or knockdown of PDLIM5. (**E**) The immunofluorescence pictures and myotube area statistics of SMSCs after overexpression or knockdown of PDLIM5. In the picture, red represents myosin and blue represents stained nuclei. * *p* < 0.05; ** *p* < 0.01 vs. NC and pcDNA3.1 (negative control) (*n* = 9).

Next, we tested the role of PDLIM5 in SMSC differentiation. Our results show that PDLIM5 overexpression increased the mRNA expression of myosin heavy chain (MyHC), myogenic differentiation 1 (MyoD1), and myogenin (MyoG), but these expressive abundances reduced in PDLIM5-knockdown cells (Figure 3A,B). In addition, the effect of PDLIM5 on MyoG and MyHC protein expression was similar to that of their corresponding genes (Figure 3C,D). At the same time, our immunofluorescence analysis showed that overexpression of PDLIM5 promoted myosin expression and induced myotube formation, but the reversed effect was caused by PDLIM5 interference (Figure 3E). Collectively, these results indicate that PDLIM5 promotes chicken SMSC differentiation.

### 3.4. Gene Expression Analysis of PDLIM5 Silenced Cells

**Figure 4:**
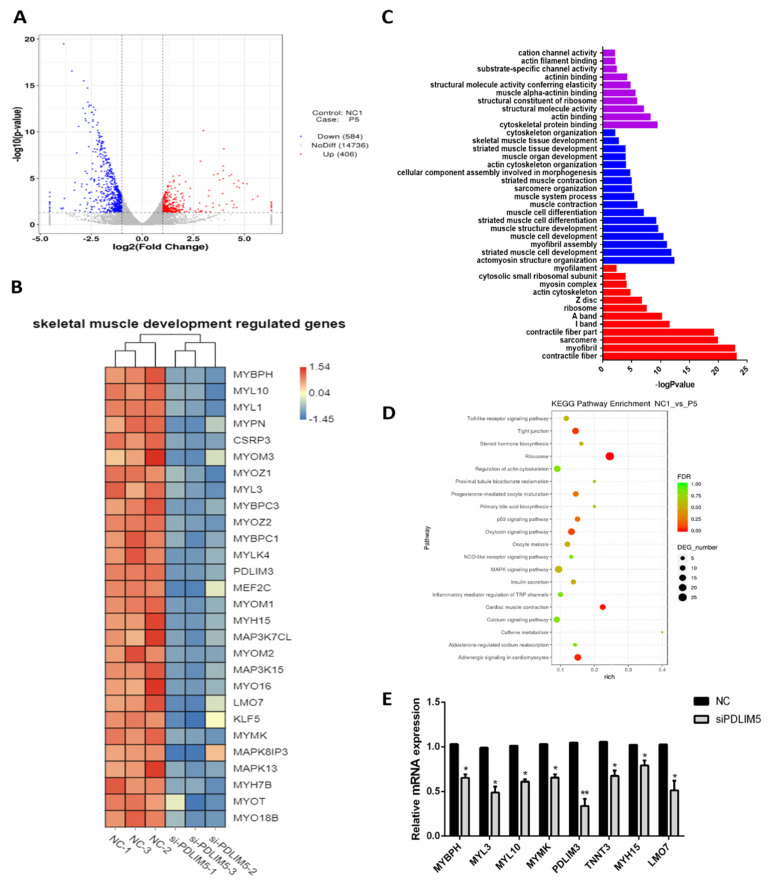
Interfering with transcriptome sequencing of si-PDLIM5 and negative control cells. (**A**) Volcano plot of statistically up-regulated or down-regulated genes after transcriptome analysis. (**B**) A heat map obtained by clustering analysis of differentially expressed genes that related to muscle development. (**C**) Analysis of differentially expressed genes by GO enrichment. (**D**) Analysis of the related pathways by KEGG enrichment. (**E**) Verification of mRNA expression of muscle development-related genes in transfected si-PDLIM5 or control. * *p* < 0.05; ** *p* < 0.01 relative to NC (negative control) (*n* = 9).

Subsequently, RNA-seq analysis was performed on cells that transfected with si-PDLIM5 and control (NCBI accession number: GSE151450). There were 990 differentially expressed genes between the PDLIM5 interference and the control groups. Among them, 406 genes up-regulated and 584 genes down-regulated (Figure 4A). In the knockdown group, the expression levels of skeletal muscle differentiation marker genes, such as myosin heavy chain 15 (MYH15), myocyte enhancer factor 2C (MEF2C), myozenin 1 (MYOZ1), and myosin light chain 10 (MYL10), were significantly down-regulated (Figure 4B). Gene ontology analysis of differential genes showed that knocking out the expression of PDLIM5 enriched functions related to muscle development, such as muscle formation, muscle contraction, muscle cell differentiation, cytoskeleton protein binding, actin cytoskeleton formation, and actin binding functions (Figure 4C). The results of the KEGG pathway indicate that these differential genes were enriched in 193 pathways, 13 of which were significantly enriched (*p* < 0.05). Among them, the oxytocin signaling pathway, p53 pathway, and MAPK pathway are strongly associated with the development of muscle (Figure 4D). Especially for the MAPK pathway, the results in Figure 4B show that the expression of MAPK13 (p38-MAPK) decreases after knocking down the expression of PDLIM5. Subsequently, we verified the expression of altered genes in RNA-seq by qPCR. After knocking down PDLIM5, the expression of these genes decreased. The trend is consistent with the transcriptome results, and the verification found that the transcriptome sequencing results are credible (Figure 4E).

### 3.5. PDLIM5 Regulated the SMSCs via p38-MAPK Signaling Pathway

**Figure 5:**
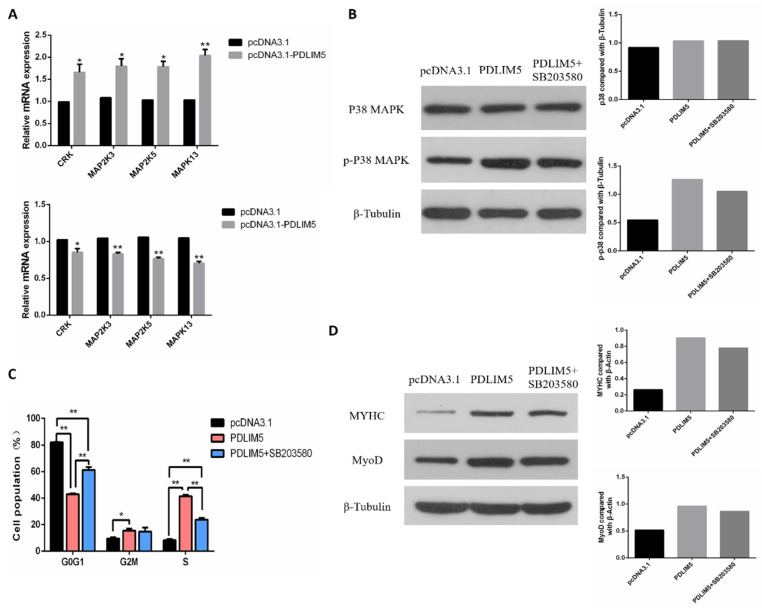
PDLIM5 regulated muscle development through the p38-MAPK pathway. (**A**) mRNA expression of MAPK pathway related genes transfected with si-PDLIM5 or pcDNA3.1-PDLIM5. (**B**) After overexpressing PDLIM5 in SMSCs and adding exogenous PDLIM5 and SB203580, the protein abundance of p38 and *p*-p38 was measured by Western blotting. In SMSCs with and without SB203580, the effect of PDLIM5 on cell proliferation (**C**) and differentiation (**D**) was determined. * *p* < 0.05; ** *p* < 0.01 vs. NC and pcDNA3.1 (negative control) (*n* = 9).

According to the results of the transcriptome, it was found that after knocking down PDLIM5, the expression of MAPK13 (p38-MAPK) decreased, and KEGG was enriched in the MAPK signaling pathway. Therefore, we then explored whether PDLIM5 regulated the development of SMSCs through the p38-MAPK pathway. First, we verified the differentially expressed MAPK pathway genes in transcriptome sequencing. The qPCR results showed that gene expression changes were consistent with transcriptome results (Figure 5A). After overexpression of PDLIM5, the total p38 protein (t-p38) and phosphorylated p38 protein (*p*-p38) were measured through Western blot. The *p*-p38 protein expression was increased, while the level of t-p38 remained unchanged. However, after adding a specific p38-MAPK inhibitor (SB203580) to PDLIM5-transfected cells, the activation of *p*-p38 was alleviated (Figure 5B). After adding exogenous PDLIM5, the ratio of G0/G1 phase cells decreased and increased in S phase, and the protein abundance of MyHC and MyoD increased. However, after adding SB203580 to PDLIM5 transfected cells, these effects were reduced (Figure 5C,D). These results indicate that PDLIM5 regulates SMSC proliferation and differentiation via the p38-MAPK signaling pathway.

## 4. Discussion

PDLIM5 is highly expressed in mouse skeletal muscle, and PDLIM5 can regulate mouse myogenesis. Our results in Figure 1 show that PDLIM5 is highly expressed in chicken skeletal muscle and heart tissue. Therefore, we speculate that PDLIM5 plays a regulatory role in chicken skeletal muscle. However, due to differences in species, specific functions need to be verified in practice. SMSCs are the primary cells of chicken muscle. After activation, SMSCs become chicken myoblasts. Therefore, in this study, we used SMSCs as an in vitro model to explore chicken muscle development and to clarify the function of PDLIM5 in the proliferation and differentiation of SMSCs. This is the first study to showcase the role of PDLIM5 in the development of chicken skeletal muscle cells. In particular, we further explored the molecular mechanism of PDLIM5 regulating the proliferation and differentiation of SMSCs through transcriptome sequencing. It provides a reference for the subsequent study of the role of PDLIM5 and even PDZ and LIM domain family genes in the process of skeletal muscle formation.

Our results show that PDLIM5 can promote the proliferation of chicken SMSCs by detecting the abundances of proliferation-related genes as well as the viability and proliferation rate of SMSCs after modulating the expression of PDLIM5 in cells. This effect of PDLIM5 on cell proliferation was similar to that recorded in numerous reports. For example, Wei et al. concluded that in human B-CPAP cells, knocking out PDLIM5 inhibited cell proliferation [20] Liu et al. reported that the high expression level of PDLIM5 promoted the occurrence and migration of prostate cancer cells [21]; and Li et al. showed that PDLIM5 can promote the proliferation of gastric cancer cells [9]. Our results show that the effects of PDLIM5 on proliferation and differentiation are uniform. Generally, cell differentiation proceeds after the end of proliferation (after the cell cycle exits). Therefore, an increase in cell proliferation will cause more cells to participate in the later differentiation, leading to an increase in cell differentiation. This means that PDLIM5 can be a positive factor in chicken myogenesis, the same as KLF4 [22], IGF1 [23], and IGF2 [24].

The formation of skeletal muscle is regulated by muscle regulatory factors, including MyoD and MyoG, which can activate muscle fiber formation and control the transcription of muscle-specific genes [25]. In addition, MyHC is a differentiation marker gene for the movement of muscles and muscle molecules, forming the skeleton of thick sarcomere [26]. In this study, knocking down of PDLIM5 could significantly inhibit the expression level of mRNA and protein of differentiation markers; meanwhile, down-regulating PDLIM5 could prevent myotube formation. As we expected, the opposite effect on SMSCs occurred in PDLIM5-overexpressed cells. The above results suggest that PDLIM5 promotes the differentiation of chicken SMSCs. Our results are similar to the research results of Ito et al., who found that ENH1 expression is significantly increased during C2C12 cell differentiation. Meanwhile, the overexpression of ENH1 resulted in up-regulation of MyoD and MyoG, and knocking down of ENH1 significantly reduced MyoD expression in C2C12 cells [27,28]. In addition, Qiu et al. found that ENH1 could be used as a target of miR-17~92 in myoblasts and the silencing of ENH1 repressed myogenic differentiation, which is also consistent with our findings. Furthermore, Qiu et al. found that ENH1 could bind to Id1 [29]. The Id protein has been shown to regulate cell proliferation and differentiation. Therefore, ENH1 participates in myogenesis by isolating Id1. This is an area that can be further studied in relation to chicken SMSCs in the future.

In the results of transcriptome sequencing, we observed that most of the differentially expressed genes between si-PDLIM5 cells and control cells were related to muscle development. This suggests that PDLIM5 has a positive effect on skeletal muscle cells differentiation. The GO (Gene Ontology) enrichment analysis of differential expression genes revealed that these genes function in the development of skeletal muscle tissue and organs, as well as in the development and contraction of striated muscle cells and myofibril assembly, which supports our speculation.

In addition, the KEGG pathway analysis showed that the differential expression genes were involved in the signaling pathway of MAPK, P53 and calcium. The MAPK signaling pathway attracted our attention since it was observed to play a major role in the regulation of the development skeletal muscle [30]. JNK, ERK, and p38 are the main members of the family that belongs to the MAPK signaling pathway [31]. Subsequently, the RT-PCR results of the key genes of the MAPK signaling pathway in PDLIM5-konckdown cells indicate that PDLIM5 may regulate the MAPK signaling pathway. Finally, we used the specific p38-MAPK inhibitor to confirm that PDLIM5 mediated the MAPK pathway to control SMSC development. In addition, PDZ and LIM domain 3 (PDLIM3) also belongs to the PDZ and LIM domain family as with PDLIM5. We found that PDLIM3 is the target gene of gga-miRNA-3525, and gga-miRNA-3525 can target PDLIM3 to inhibit the development of chicken SMSCs through the p38-MAPK pathway [32]. In addition, four and a half LIM domain protein 2 (FHL2) regulates the expression of interleukin-6 in muscle cells through the p38-MAPK signaling pathway [33]. FHL2 interacts with LC3 during the formation of autophagosomes to regulate muscle cell development [34].

## 5. Conclusions

**Figure 6:**
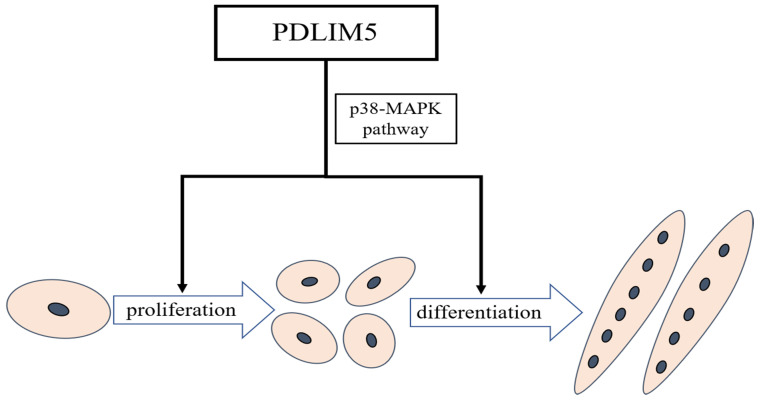
Schematic diagram of PDLIM5 regulating chicken skeletal muscle satellite cell proliferation and differentiation.

In summary, we have demonstrated that PDLIM5 can promote chicken SMSC proliferation and differentiation. Transcriptome sequencing found that knocking down PDLIM5 can reduce the expression of many muscle-development-related genes, such as MYBPH, MYH15, and MEF2C. In addition, we further verified that PDLIM5 can promote muscle development through the p38-MAPK pathway (Figure 6). These results can help us to detect more functions of PDLIM5 and even genes containing PDZ and LIM domains in myogenesis.

## Figures and Tables

**Table 1:** The sequences of RNA oligonucleotide and plasmid.

Name	Sequence (5′–3′)
Si-PDLIM5	GGAUAAGUCAGACGGGAUTT AUCCCGUCUGUACUUAUCCTT
Si-NC	UUCUCCGAACGUGUCACGUTT ACGUGACACGUUCGGAGAATT

## Data Availability

The data presented in this study are available on request from the corresponding author.

## Supplementary Materials

The following are available online at https://www.mdpi.com/article/10.3390/ani11041016/s1, Table S1: Primers used for quantitative real-time PCR, Table S2: Information on antibodies used in Western blotting.
