# Supplementary material for: PDLIM5 Affects Chicken Skeletal Muscle Satellite Cell Proliferation and Differentiation via the p38-MAPK Pathway
Source: Animals (Basel). 2021 Apr 4;11(4):1016. doi: 10.3390/ani11041016 (PMC8065989; doi:10.3390/ani11041016)
Supplement: Supplementary file 1 [file animals-11-01016-s001.pdf]

Table S1. Primers used for quantitative real-time PCR

Gene	Primer Sequences (5′-3′)	Product size (bp)	TM(℃)
β-actin	F: GTCCACCGCAAATGCTTCTAA<br>R: TGCGCATTTATGGGTTTTGTT	78	58
MyoG	F: CGTGTGCCACAGCCAATG<br>R: CCGCCGGAGAGAGACCTT	63	60
MyoD1	F: GCCGCCGATGACTTCTATGA<br>R: CAGGTCCTCGAAGAAGTGCAT	66	60
MYHC	F: GAAGGAGACCTCAACGAGATGG<br>R: ATTCAGGTGTCCCAAGTCATCC	138	60
CCND1	F: CTCCTATCAATGCCTCACA<br>R: TCTGCTTCGTCCTCTACA	165	54
PCNA	F: AACACTCAGAGCAGAAGAC<br>R: GCACAGGAGATGACAACA	225	55
CDK2	F: CCAGAACCTCCTCATCAAC<br>R: CAGATGTCCACAGCAGTC	171	55
PDLIM5	F：GGCTGATTGAAGATACTGAAG<br>R：GGTGCTGAAGGAGATGTG	259	58
PDLIM3	F: AAGCACCTGTAACAAAGATA<br>R: GCCCTCCACAAAGAAGTAGC	188	55
MYBPH	F: ATCCGCCTACCTCGTCAG<br>R: GGCTGGTTGTCCTTGGTC	122	58
MYL10	F: TGTTGCTTAACCTCTTGCTTT<br>R: TACCAAATGCTCTTCCCAGT	89	56
MYL3	F: GAAGAACCCAAACCAGCA<br>R: CCCAAAGCCCTCAAGAC	200	55
TNNT3	F: GGCTGAGAAGGAGAAGGAG<br>R: GCTGTATGAGGCACCCA	136	56
MYMK	F: TCCCCACCATCAGCATC<br>R: GCATGAAACATAGCACCGA	129	56
MAPK13	F: TTCCTGAGTCGTGTTTGGT<br>R: GGGGCATGGCTGTAGTAA	145	56

CAV3	F: GTGCCCTGCATCAAGAG<br>R: CGCAGCATAACCCTGAC	134	55
CRK	F: CACTCCGCTCCCTAACC<br>R: CCTTCCCACTGACCACTC	150	56
MAP2K3	F: GCCTATGGTGTGGTGGAGA<br>R: AAGCAGTCAACCGTCCTCA	143	58
MAP2K5	F: GGCCAGATGAATGAACAAG<br>R: GCCAGGATTTTCCCACTA	104	54

Table S2. Information on antibodies used in Western blotting

Antibody	Company	Dilution ratio
anti-MYHC	Santa Cruz Biotechnology, USA (sc-32732)	1:200
anti-MyoD	Santa Cruz Biotechnology, USA (sc-377460)	1:500
anti-phospho-p38 MAPK	Sigma, USA (SAB4504497)	1: 200
anti-p38 MAPK	Sigma, USA (SAB4500491)	1:200
anti-β-Tubulin	ZENBIO, Beijing, China (700608)	1: 5,000
